# Supplementary material for: Accuracy of perceived glaucoma risk by patients in a clinical setting
Source: PLoS One. 2021 Sep 16;16(9):e0257453. doi: 10.1371/journal.pone.0257453 (PMC8445404; doi:10.1371/journal.pone.0257453)
Supplement: S1 File — Questionnaire used in the current study. (PDF) [file pone.0257453.s001.pdf]

問卷版本：第一版
西元 2018 年 7 月 31 日

問卷編號	樣本序號	總樣本數
		2500

眼疾之治療與認知: 病患因素與區域資源配置

施測地點：____________(此欄由訪員填寫)

請問您是否是病患本人? □是 □否（如填「否」，請問您與病患的關係：______________）
*如果您不是病患本人，麻煩您以下所有問題均以病患之狀況填答。

第一部分：基本資料與個人病史

A1 性　　別：□男 □女

A2 您的年齡：________ 歲

A3 教育程度：□不識字 □小學或以下 □國中 □高中／高職 □大學 □碩士 □博士

A4 婚姻狀況：□未婚 □已婚/同居 □喪偶

A5 您目前居住的縣市：______________ 縣/市

A6 您的身高：________公分

A7 您的體重：________公斤

A8 您是否吸菸？□從未吸菸 □目前吸菸 □已戒菸

A9 一般而言，你對目前的健康狀況是？□極好的 □很好的 □好 □普通 □不好

A10 如果您想看眼科，從您居住的地方到最近一家您信任的眼科醫院或診所大約需要多久(用您習慣的交通工具到達)? _________分鐘

A11 您自評家中經濟狀況：□許多結餘 □些許結餘 □收支平衡 □些許不足 □嚴重不足

A12 您目前工作狀態：□有工作(有薪水或酬勞) □無工作，但目前在找有薪水或酬勞的工作 □無工作也無在找工作/退休

A13 請問您的運動習慣 (定義:一次 15 分鐘以上刻意以運動為目的之活動):
□無運動 □每週 1~2 次 □每週 3~4 次 □每週 5 次以上

A14 您是否有下列疾病 (經醫師確診)?
□癌症 □糖尿病 □高血壓 □高血脂 □貧血
□心臟病 □肺臟疾病 □腎臟病 □肝臟疾病 □曾經中風 □動脈硬化
□老年失智 □巴金氏症 □憂鬱症 □思覺失調症

A15 您目前經眼科醫師確診的眼睛疾病：
□高度近視(500 度以上) □高度遠視(300 以上) □高度散光(250 以上) □弱視 □斜視
□圓錐角膜 □角膜疤痕或退化 □白內障未手術(單眼) □白內障未手術(雙眼)
□青光眼(單眼) □青光眼(雙眼) □黃斑部病變 □曾經視網膜剝離
□糖尿病視網膜病變 □色素性視網膜炎(夜盲症) □視神經萎縮

A16 是否曾接受過以下眼科手術?

☐白內障手術 ☐視網膜手術 ☐角膜手術 ☐青光眼手術 ☐眼球外傷手術

☐其他(請註明)__________

A17 您上次量眼壓是多久以前?

☐本次就診 ☐______月前 ☐不知道

A18 就您所知，您最近一次眼壓測量之眼壓為何?

右眼________ mmHg 左眼 ________ mmHg ☐不知道

A19 您是否有定期去醫療院所檢查青光眼的習慣(指特別要求醫師幫您檢查青光眼，或是知道醫師有幫您檢查)?

☐一年一次 ☐半年一次 ☐無特別習慣

A20 您過去半年大約看過幾次眼科門診(醫院或診所)? _______ 次

A21 您上一次看眼科大約是多久前? 約_______月前

A22 您是否有家族青光眼病史?

☐父親 ☐母親 ☐祖父母 ☐兄弟姊妹 ☐不知道

A23 您認為您在未來一年罹患青光眼的機率是否很高?

☐很高 ☐中等 ☐低 ☐完全沒概念

A24 您認為您在未來一年罹患青光眼的機率跟您相同年齡的人比是否比較高?

☐比較高 ☐一樣 ☐比較低 ☐完全沒概念

A25 您自評您的眼睛健康程度跟您相同年齡的人比:

☐比較好 ☐差不多 ☐比較差

A26 您目前的眼睛健康程度是否讓您感到焦慮?

☐非常焦慮 ☐有一點焦慮 ☐不焦慮

～後面還有～

第二部分:視力相關問題

一、基本問題

B1 您認為您目前的兩眼並用視力（如有眼鏡或隱形眼鏡，以戴上眼鏡或隱形眼鏡而言）屬於極佳、好、普通、不好、非常不好、或完全眼盲？

☐極佳 ☐好 ☐普通 ☐不好 ☐非常不好 ☐完全眼盲

B2 您多常擔心您的視力？

☐從不擔心 ☐很少擔心 ☐有時候擔心 ☐經常擔心 ☐總是擔心

B3 您的眼睛或其四周曾感到何種程度的疼痛或不適（例如灼熱、發癢、疼痛）？您認為該程度屬於下列那一選項：

☐從未有過 ☐輕微 ☐中等 ☐劇烈 ☐非常劇烈

二、做事情時的困難度(單選)

如感到困難，是何種程度的困難？（假如您是需要帶著眼鏡或隱形眼鏡做這些事情，

以下問題為戴眼鏡或隱形眼鏡下的狀況）

B4 閱讀一般報紙文字，您會感到何種程度的困難？您認為困難度屬於下列那一選項：

☐完全無困難 ☐有點困難 ☐中等困難 ☐極度困難

☐因視力之故不再閱讀 ☐因其他緣故不再閱讀、沒興趣閱讀或不識字

B5 在做需要近距離看清楚的工作或嗜好活動時，例如在家煮飯、縫衣、修理東西或使用工具，您會感到何種程度的困難？您認為困難度屬於下列那一選項：

☐完全無困難 ☐有點困難 ☐中等困難 ☐極度困難

☐因視力之故不再做 ☐因其他緣故不再做或沒興趣做

B6 在放滿物品的架子上找東西，您會因視力之故感到何種程度的困難？

☐完全無困難 ☐有點困難 ☐中等困難 ☐極度困難

☐因視力之故不再找 ☐因其他緣故不再尋找或沒興趣找

B7 看街道路牌或商店名稱，您會感到何種程度的困難？

☐完全無困難 ☐有點困難 ☐中等困難 ☐極度困難

☐因視力之故不再看 ☐因其他緣故不再看或沒興趣看

B8 在光線昏暗或夜晚時走下台階、樓梯、路邊石階，您會因視力之故感到何種程度的困難？

☐完全無困難 ☐有點困難 ☐中等困難 ☐極度困難

☐因視力之故不再走 ☐因其他緣故不再走或沒興趣走

B9 行走時注意沿路旁邊的東西，您會因視力之故感到何種程度的困難？

☐完全無困難 ☐有點困難 ☐中等困難 ☐極度困難

☐因視力之故不再注意 ☐因其他緣故不再注意或沒興趣注意

B10 觀看人們對您說的話如何反應，您會因視力之故感到何種程度的困難？

☐完全無困難 ☐有點困難 ☐中等困難 ☐極度困難

☐因視力之故不再觀看 ☐因其他緣故不再觀看或沒興趣觀看

B11 挑選並搭配您自身的衣服，您會因視力之故感到何種程度的困難？

☐完全無困難 ☐有點困難 ☐中等困難 ☐極度困難

☐因視力之故不再搭配 ☐因其他緣故不再搭配或沒興趣搭配

B12　和別人一起去人家家裡、宴會或餐廳作客，您會因視力之故感到何種程度的困難？
□完全無困難　□有點困難　□中等困難　□極度困難
□ 因視力之故不再去　□因其他緣故不再去或沒興趣去

B13　出去看電影、戲劇、運動比賽，您會因視力之故感到何種程度的困難？
□完全無困難　□有點困難　□中等困難　□極度困難
□因視力之故不再去　□因其他緣故不再去或沒興趣去

以下是有關開車的問題：

B14　您目前會至少偶爾開車嗎？
□從未開過車(跳至第三部分問題 17)
□否(不會)(跳至 14-1)
□是(會)(跳至 14-3)

B14-1　如答案為否，則請問：您從未開過車或您已放棄開車？
□從未開過車(跳至第三部分問題 17)
□放棄開車(跳至 14-2)

B14-2　如答案為**放棄開車**，則請問：放棄開車主要是因為您的視力嗎？或主要是因其他緣故？或因您的視力與其他緣故兩者皆有？
□主要因視力(跳至第三部分問題 17)
□主要因其他緣故(跳至第三部分問題 17)
□兩者皆有(跳至第三部分問題 17)

B14-3　如您**目前開車**，則請問：白天開在熟悉的地方，您會感到何種程度的困難？您認為困難度屬於下列那一選項：
□完全無困難　□有點困難　□中等困難　□極度困難

B14-4　在艱難的狀況下開車，例如天候不佳、尖峰時段、在高速公路上、或在市區交通裡，您會感到何種程度的困難？您認為困難度屬於下列那一選項：
□完全無困難　□有點困難　□中等困難　□極度困難
□因視力之故已不再在艱難狀況下開車
□因其他緣故不再在艱難狀況下開車或沒興趣在艱難狀況下開車

B15　晚上開車，您會感到何種程度的困難？您認為困難度屬於下列那一選項：
□完全無困難　□有點困難　□中等困難　□極度困難
□因視力之故已不再晚上開車
□因其他緣故已不再晚上開車或沒興趣晚上開車

～後面還有～

三、視力問題的答覆

下面問題是有關您的視力如何影響您做的事情。請以總是、經常、有時候、很少、從不回答下列各問題。（請每題勾選一項）

		1 總是	2 經常	3 有時	4 很少	5 從不
C1	您因視力之故完成的比期望完成的少嗎？	☐	☐	☐	☐	☐
C2	您因視力之故在工作時或做其他活動方面受到限制嗎？	☐	☐	☐	☐	☐
C3	您眼睛或其四周的疼痛或不適(例如灼熱、發癢、疼痛）影響您不能做想做的事情之程度為何？	☐	☐	☐	☐	☐

請以完全符合事實、大致符合事實、大致不符事實、完全不符事實、或不確定回答下列各陳述。（請每題勾選一項）

		1 完全符合事實	2 大致符合事實	3 不確定	4 大致不符合事實	5 完全不符合事實
C4	因視力之故，我經常在家不出門	☐	☐	☐	☐	☐
C5	因視力之故，我經常感到沮喪	☐	☐	☐	☐	☐
C6	因視力之故，我對所做的事情無法好好控制	☐	☐	☐	☐	☐
C7	因視力之故，我必須過度依賴別人告訴我該怎麼做	☐	☐	☐	☐	☐
C8	因視力之故，我需要許多別人的協助	☐	☐	☐	☐	☐
C9	因視力之故，我擔心做出使自己或使別人尷尬的事情	☐	☐	☐	☐	☐

第三部分:醫療接受度與相關知識

以下我們想知道您對白內障和青光眼的相關知識。

D1 請問下列何種是治療白內障和青光眼的方法?(對的打勾)

**白內障**	**青光眼**
☐戴眼鏡	☐戴眼鏡
☐手術	☐手術
☐雷射治療	☐雷射治療
☐定期看眼科	☐定期看眼科
☐特殊維他命或補品	☐特殊維他命或補品
☐中草藥	☐中草藥
☐口服西藥	☐口服西藥
☐眼藥水	☐眼藥水
☐不知道	☐不知道

D2 請問下列何種是白內障和青光眼的症狀?(對的打勾)

**白內障**	**青光眼**
☐視力模糊	☐視力模糊
☐眼睛痛	☐眼睛痛
☐光線周圍看起來有虹霞	☐光線周圍看起來有虹霞
☐噁心	☐噁心
☐頭痛	☐頭痛
☐看東西色調改變	☐看東西色調改變
☐怕光	☐怕光
☐眼前黑點	☐眼前黑點
☐複視	☐複視
☐不知道	☐不知道

D3	青光眼可以根治。	☐對	☐錯	☐不知道
D4	白內障會造成青光眼。	☐對	☐錯	☐不知道
D5	用眼過度會造成青光眼。	☐對	☐錯	☐不知道
D6	無家族病史則罹患青光眼的機率較低。	☐對	☐錯	☐不知道
D7	青光眼初期不會有症狀。	☐對	☐錯	☐不知道
D8	一般而言眼壓低於 30mmHg 屬於正常範圍。	☐對	☐錯	☐不知道

D9 請問下列何者是造成青光眼的危險因素?

年齡/老化	☐是	☐否	☐不知道
遺傳	☐是	☐否	☐不知道
高眼壓	☐是	☐否	☐不知道
高度近視或遠視	☐是	☐否	☐不知道
眼睛外傷	☐是	☐否	☐不知道
長期使用類固醇	☐是	☐否	☐不知道
糖尿病	☐是	☐否	☐不知道
偏頭痛	☐是	☐否	☐不知道
高血壓	☐是	☐否	☐不知道
肥胖	☐是	☐否	☐不知道
缺乏運動	☐是	☐否	☐不知道
營養不均衡	☐是	☐否	☐不知道
看太多手機腦電腦電視等 3C 產品	☐是	☐否	☐不知道
睡眠不足	☐是	☐否	☐不知道

<u>第四部分：眼鏡配戴狀況</u>

E1 請問您目前是否**<u>擁有</u>**具矯正視力功能的眼鏡或隱形眼鏡?

☐是(回答 E2)

<table>
<tr><td>E2</td><td colspan="3">☐擁有眼鏡（非隱形眼鏡）
⬇（回答E3~E5）
☐擁有隱形眼鏡
⬇（回答E6~E8）</td></tr>
<tr><td>E3</td><td>戴眼鏡的原因（可複選）☐近視
☐遠視 ☐老花 ☐散光 ☐其他________
請問您配戴眼鏡多久了? ______年</td><td>E6</td><td>戴隱形眼鏡的原因（可複選）☐近視
☐遠視 ☐老花 ☐散光 ☐其他________
請問您配戴隱形眼鏡多久了? ______年</td></tr>
<tr><td>E4</td><td>您每天戴<u>眼鏡</u>的頻率(指清醒時)
☐大部分時間 ☐有時 ☐偶爾 ☐幾乎不戴</td><td>E7</td><td>您每天戴<u>隱形眼鏡</u>的頻率(指清醒時)
☐大部分時間 ☐有時 ☐偶爾 ☐幾乎不戴</td></tr>
<tr><td>E5</td><td>下列何者是影響您戴眼鏡或隱形眼鏡頻率的因素（可複選)?
☐戴眼鏡時會頭暈或不舒服
☐感覺配戴了眼視力進步不大
☐戴眼鏡太麻煩
☐視力在日常生活沒問題
☐眼鏡壞了或遺失</td><td>E8</td><td>下列何者是影響您戴隱形眼鏡頻率的因素(可複選)?
☐戴隱形眼鏡時會頭暈或不舒服
☐感覺配戴了眼視力進步不大
☐戴隱形眼鏡太麻煩
☐視力在日常生活沒問題
☐隱形眼鏡壞了或遺失</td></tr>
</table>

☐否(回答 E9)

E9	之前是否有眼科醫師或驗光人員建議您配眼鏡或隱形眼鏡?<br>☐是（第一次是多久以前? _______）(續答下題)<br>☐否（請回答下部分F1）
E10	請問您沒配眼鏡或隱形眼鏡的原因（可複選）<br>☐ 覺得視力夠用，暫時不需要配眼鏡<br>☐ 沒時間去配眼鏡/太麻煩<br>☐ 和家人／朋友共用即可<br>☐ 眼鏡太貴<br>☐ 預期配了眼鏡視力也不會進步太多<br>☐ 預期戴眼鏡會不舒服<br>☐ 美觀問題<br>☐ 眼鏡選項太多無法理解<br>☐ 有配過，但眼鏡壞了或遺失

F1 之前(至少半年以上)是否有眼科醫師(不論在醫院或診所)建議您提早接受白內障手術?

☐是，我有進行手術(問卷結束)
☐是，但我沒有進行手術(請繼續回答F2~F13)
☐否，因為沒看眼科/那時沒有白內障(問卷結束)
☐否，因為醫師說我的白內障還沒嚴重到需要開刀(問卷結束)

我們想知道您當時沒有接受白內障手術的原因?

		1 是重要因素	2 算是重要因素	3 不是重要因素
F2	害怕手術失敗造成失明。	☐	☐	☐
F3	即使手術不造成失明，我擔心手術造成後遺症或是白內障復發。	☐	☐	☐
F4	沒時間做手術/家人沒時間陪伴做手術。	☐	☐	☐
F5	擔心復原時間太長會影響生活/工作。	☐	☐	☐
F6	我並不知道自己有白內障。	☐	☐	☐
F7	我認為我的白內障狀況還沒嚴重到需要開刀。	☐	☐	☐
F8	白內障手術太多種我無法理解，或是沒時間研究。	☐	☐	☐
F9	我想透過其他非手術的辦法治療白內障<br>(例如點眼藥水、吃草藥、減少晒太陽等)。	☐	☐	☐
F10	害怕會痛。	☐	☐	☐
F11	覺得開刀後視力也不會進步或進步幅度不大。	☐	☐	☐
F12	擔心手術會需要花不少自費的費用。	☐	☐	☐
F13	其他__________________	☐	☐	☐

問卷結束。非常謝謝您的回答，請交與您的醫師或護理人員。

To be filled by physician or medical staff

病歷號:____________ **填表日期：____年___月___日**

1. 該病患確診眼疾：

☐ 高度近視(500 度以上) ☐高度遠視(300 以上) ☐高度散光(250 以上) ☐弱視 ☐斜視
☐ 圓錐角膜 ☐角膜疤痕或退化
☐ 白內障(單眼) ☐ 白內障(雙眼) ☐ 青光眼(單眼) ☐ 青光眼(雙眼)
☐ 黃斑部病變 ☐曾經視網膜剝離 ☐糖尿病視網膜病變 ☐色素性視網膜炎(夜盲症)
☐視神經萎縮

2. 是否曾接受過以下眼科手術?

☐白內障手術 ☐視網膜手術 ☐角膜手術 ☐青光眼手術 ☐眼球外傷手術 ☐其他(請註明)_________

3. 此病患本次就診是否有量眼壓? ☐有 ☐無

4. 該病患最近一次(或此次)_眼壓測量之眼壓為何? ________ mmHg ☐不知道

5. Does this patient need cataract surgery right now?

☐Yes, and already delayed
☐Yes, not delayed
☐No

6. Physician-rated risk of glaucoma:

☐ High
☐ Medium
☐ Low
☐ Don’t know
☐The patient already has glaucoma.
☐left eye ☐right eye ☐both eyes

7. Please given an overall assessment of the risk based on the following risk factors, please check the boxes:

☐are over age 40
☐have family members with glaucoma
☐have high eye pressure
☐are farsighted or nearsighted
☐have had an eye injury
☐use long-term steroid medications
☐have corneas that are thin in the center
☐have thinning of the optic nerve
☐have diabetes, and high blood pressure

8. How many times have you seen this patient?

☐ First time ☐ 2-3
☐ 4-5 ☐ >=5

醫師簽名：__________________

問卷結束。非常謝謝您的回答，請交與您的醫師或護理人員。
