# Supplementary material for: Accuracy of perceived glaucoma risk by patients in a clinical setting
Source: PLoS One. 2021 Sep 16;16(9):e0257453. doi: 10.1371/journal.pone.0257453 (PMC8445404; doi:10.1371/journal.pone.0257453)
Supplement: S2 File — Questionnaire used in the current study. (DOCX) [file pone.0257453.s002.docx]

| Questionnaire No. | Subject No. | Total number  of subjects |
| --- | --- | --- |
|  |  |  |

***The original questionnaire was in Chinese. Below is a translation of the original version. The format may vary from the original version. All rights reserved.**

　　　　　　　　　Test location： _ (filled in by the interviewer)

**Are you the patient?** □Yes □No (IF NO, what is your relationship with the patient: _______________)

***** If you are not the patient, please answer all the following questions based on the patient's condition.

##

## Part 1: Basic information and personal medical history

| A1 | Gender: □Male □Female |
| --- | --- |
| A2 | Age: years old |
| A3 | Level of education: □Illiterate □Elementary school or below □Junior high school  □High school/High vocational □Bachelor degree □Master degree □Doctoral degree |
| A4 | Marital status: □Never married □Married/Cohabiting □Widowed/Other |
| A5 | City of residence: County /City |
| A6 | Hight: cm |
| A7 | Weight: kg |
| A8 | Do you smoke? □Never smoked □Current smoker □Quitted |
| A9 | In general, would you say your overall health is: □Excellent □Very good □Good □Fair □Poor |
| A10 | How long does it take for you to go from where you live to the nearest ophthalmologist you trust? (by the means of transportation you are used to take)  __________minutes |
| A11 | What’s your family's financial situation? □Lots of surpluses □Some surpluses □Balanced □Slightly shortages □Seriously shortages |
| A12 | Employment status □Have a paid job □Not having a job, but currently looking for a paid job  □Not having a job and are not looking for a job / Retired |
| A13 | Exercise frequency (Definition of exercise: an activity that deliberately aims at sports for more than 15 minutes): □No exercise □1~2 times a week □3~4 times a week  □5 times a week or more |
| A14 | Do you have the following diseases (diagnosed by a physician)?  □Cancer □Diabetes □High blood pressure □Hyperlipidemia □Anemia  □Heart disease □Lung disease □Kidney disease □Liver disease □Have a stroke  □Arteriosclerosis □Senile Dementia □Parkinson's disease □Depression □Schizophrenia |
| A15 | Do you currently have the following eye diseases diagnosed by an ophthalmologist?  □High myopia (above 500 degrees) □High hyperopia (above 300)  □High astigmatism (above 250)  □Amblyopia □Strabismus □Keratoconus □Corneal scars or degeneration  □Cataract without surgery (one eye) □Cataract without surgery (both eyes)  □Glaucoma (one eye) □Glaucoma (both eyes) □Macular disease □Retinal detachment  □Diabetic retinopathy □Retinitis pigmentosa (night blindness) □Optic nerve atrophy. |

| A16 | Have you ever received the following eye surgery?  □Cataract surgery □Retina surgery □Cornea surgery □Glaucoma surgery  □Eyeball trauma surgery □Others (please specify) |
| --- | --- |
| A17 | When was the last time you measured your intraocular pressure?  □During this visit □ __months ago □Don’t know |
| A18 | Do you know the intraocular pressure of your last intraocular pressure measurement?  Right eye mmHg Left eye mmHg □Don’t know |
| A19 | Do you go to hospitals or clinics to check whether you have glaucoma regularly? (Meaning specifically requesting the doctor to check for whether you have glaucoma, or knowing that the doctor will do the check for you)?  □Once a year □Once every six months □No |
| A20 | How many times have you visited an ophthalmologist in the past six months (hospital or clinic)?  time(s) |
| A21 | When was the last time you visited an ophthalmologist? About months ago |
| A22 | Do you have a family history of glaucoma?  □Father □Mother □Grandparents □Siblings □Don’t know □No family history |
| A23 | Do you think you have a high chance of developing glaucoma in the next year?  □High □Medium □Low □I have no idea |
| A24 | Do you think your chance of developing glaucoma in the next year will be higher than that of someone of your age?  □Higher □The same □Lower □I have no idea |
| A25 | How would you rate your eye health compared to people of the same age:  □Better □Same □Worse |
| A26 | Do you feel anxious about your current eye health condition?  □Very anxious □A little anxious □Not anxious |

*～ PLEASE TURN THE PAGE AND CONTINUE～*

## Part 2: Vision-related question

| **1 - GENERAL HEALTH AND VISION** | |
| --- | --- |
| B1 | At the present time, would you say your eyesight using both eyes (with glasses or contact lenses, if you wear them) is excellent, good, fair, poor, or very poor or are you completely blind?  □Excellent □Good □Fair □Poor □Very poor □Completely blind |
| B2 | How much of the time do you worry about your eyesight?  □None of the time □A little of the time □Some of the time  □Most of the time □All of the time |
| B3 | How much pain or discomfort have you had in and around your eyes (for example, burning, itching, or aching)? Would you say it is:  □None □Mild □Moderate □Severe □Very Severe |
| **2 - DIFFICULTY WITH ACTIVITIES**  The next questions are about how much difficulty, if any, you have doing certain activities wearing your glasses or contact lenses if you use them for that activity. | |
| B4 | How much difficulty do you have reading ordinary print in newspapers? Would you say you have: (READ CATEGORIES AS NEEDED) |
|  | □No difficulties at all □A little difficulty □Moderate difficulty □Extreme difficulty  □Stopped doing this because of your eyesight  □Stopped doing this for other reasons or not interested in doing this |
| B5 | How much difficulty do you have doing work or hobbies that require you to see well up close, such as cooking, sewing, fixing things around the house, or using hand tools? Would you say: (READ CATEGORIES AS NEEDED) |
|  | □No difficulties at all □A little difficulty □Moderate difficulty □Extreme difficulty  □Stopped doing this because of your eyesight  □Stopped doing this for other reasons or not interested in doing this |
| B6 | Because of your eyesight, how much difficulty do you have finding something on a crowded shelf?  (READ CATEGORIES AS NEEDED) |
|  | □No difficulties at all □A little difficulty □Moderate difficulty □Extreme difficulty  □Stopped doing this because of your eyesight  □Stopped doing this for other reasons or not interested in doing this |
| B7 | How much difficulty do you have reading street signs or the names of stores?  (READ CATEGORIES AS NEEDED) |
|  | □No difficulties at all □A little difficulty □Moderate difficulty □Extreme difficulty  □Stopped doing this because of your eyesight  □Stopped doing this for other reasons or not interested in doing this |
| B8 | Because of your eyesight, how much difficulty do you have going down steps, stairs, or curbs in dim light or at night?  (READ CATEGORIES AS NEEDED) |
|  | □No difficulties at all □A little difficulty □Moderate difficulty □Extreme difficulty  □Stopped doing this because of your eyesight  □Stopped doing this for other reasons or not interested in doing this |
| B9 | Because of your eyesight, how much difficulty do you have noticing objects off to the side while you are walking along?  (READ CATEGORIES AS NEEDED) |
|  | □No difficulties at all □A little difficulty □Moderate difficulty □Extreme difficulty  □Stopped doing this because of your eyesight  □Stopped doing this for other reasons or not interested in doing this |
| B10 | Because of your eyesight, how much difficulty do you have seeing how people react to things you say?  (READ CATEGORIES AS NEEDED) |
|  | □No difficulties at all □A little difficulty □Moderate difficulty □Extreme difficulty  □Stopped doing this because of your eyesight  □Stopped doing this for other reasons or not interested in doing this |
| B11 | Because of your eyesight, how much difficulty do you have picking out and matching your own clothes?  (READ CATEGORIES AS NEEDED) |
|  | □No difficulties at all □A little difficulty □Moderate difficulty □Extreme difficulty  □Stopped doing this because of your eyesight  □Stopped doing this for other reasons or not interested in doing this |
| B12 | Because of your eyesight, how much difficulty do you have visiting with people in their homes, at parties, or in restaurants?  (READ CATEGORIES AS NEEDED) |
|  | □No difficulties at all □A little difficulty □Moderate difficulty □Extreme difficulty  □Stopped doing this because of your eyesight  □Stopped doing this for other reasons or not interested in doing this |
| B13 | Because of your eyesight, how much difficulty do you have going out to see movies, plays, or sports events?  (READ CATEGORIES AS NEEDED) |
|  | □No difficulties at all □A little difficulty □Moderate difficulty □Extreme difficulty  □Stopped doing this because of your eyesight  □Stopped doing this for other reasons or not interested in doing this |
| B14 | Now, I’d like to ask about driving a car. Are you currently driving, at least once in a while? |
|  | □Never (Skip To question C1 in part 3)  □No (Skip To 14-1)  □Yes (Skip To 14-3) |
| B14-1 | IF NO, have you never driven a car or have you given up driving? |
|  | □Never drove (Skip To question C1 in part 3)  □Gave up (Skip To 14-2) |
| B14-2 | IF GAVE UP DRIVING: Was that mainly because of your eyesight, mainly for some other reason, or because of both your eyesight and other reasons? |
|  | □Mainly eyesight (Skip To C1 in part 3)  □Mainly other reasons (Skip To C1 in part 3)  □Both eyesight and other reasons (Skip To C1 in part 3) |
| B14-3 | IF CURRENTLY DRIVING: How much difficulty do you have driving during the daytime in familiar places? Would you say you have: |
|  | □No difficulties at all □A little difficulty □Moderate difficulty □Extreme difficulty |
| B14-4 | How much difficulty do you have driving in difficult conditions, such as in bad weather, during rush hour, on the freeway, or in city traffic? Would you say you have:  (READ CATEGORIES AS NEEDED) |
|  | □No difficulties at all □A little difficulty □Moderate difficulty □Extreme difficulty  □Stopped doing this because of your eyesight  □Stopped doing this for other reasons or not interested in doing this |
| B15 | How much difficulty do you have driving at night? Would you say you have:  (READ CATEGORIES AS NEEDED) |
|  | □No difficulties at all □A little difficulty □Moderate difficulty □Extreme difficulty  □Stopped doing this because of your eyesight  □Stopped doing this for other reasons or not interested in doing this |

*～ PLEASE TURN THE PAGE AND CONTINUE～*

| **3 - RESPONSES TO VISION PROBLEMS** | | | | | | |
| --- | --- | --- | --- | --- | --- | --- |
| The questions in this section ask whether things you do are affected by your vision. For each one, I’d like you to tell me if it is true for you all, most, some, a little, or none of the time.  (Tick One On Each Line) | | | | | | |
| READ CATEGORIES: | | 1.  All of the time | 2.  Most of the time | 3.  Some of the time | 4.  A little of the time | 5.  None of the time |
| C1 | Do you accomplish less than you would like because of your vision? | □ | □ | □ | □ | □ |
| C2 | Are you limited in how long you can work or do other activities because of your vision? | □ | □ | □ | □ | □ |
| C3 | How much does pain or discomfort in or around your eyes, for example, burning, itching, or aching, keep you from doing what you’d like to be doing? Would you say: | □ | □ | □ | □ | □ |

| For each of the following statements, please tell me if it is definitely true, mostly true, mostly false, definitely false, or not sure.  (Tick One On Each Line) | | | | | | |
| --- | --- | --- | --- | --- | --- | --- |
|  |  | 1.  Definitely True | 2.  Mostly True | 3.  Not Sure | 4.  Mostly False | 5.  Definitely False |
| C4 | I stay home most of the time because of my eyesight. | □ | □ | □ | □ | □ |
| C5 | I feel frustrated a lot of the time because of my eyesight. | □ | □ | □ | □ | □ |
| C6 | I have much less control over what I do, because of my eyesight. | □ | □ | □ | □ | □ |
| C7 | Because of my eyesight, I have to rely too much on what other people tell me. | □ | □ | □ | □ | □ |
| C8 | I need a lot of help from others because of my eyesight. | □ | □ | □ | □ | □ |
| C9 | I worry about doing things that will embarrass myself or others, because of my eyesight. | □ | □ | □ | □ | □ |

| **Part 3: Medical acceptance and related knowledge**  **We would like to know your knowledge about cataracts and glaucoma.** | | | | | |
| --- | --- | --- | --- | --- | --- |
| D1 | Which of the following are the treatments of cataract and glaucoma?  (Select what you think is right) | | | | |
|  | **Cataract** | **Glaucoma** | |  | |
|  | □ Wearing glasses | □ Wearing glasses | | | |
|  | □ Surgery | □ Surgery | | | |
|  | □ Laser | □ Laser | | | |
|  | □ Visit an ophthalmologist regularly | □ Visit an ophthalmologist regularly | | | |
|  | □ Specific vitamins or supplements | □ Specific vitamins or supplements | | | |
|  | □ Chinese herbal medicine | □ Chinese herbal medicine | | | |
|  | □ Oral medication | □ Oral medication | | | |
|  | □ Eye drops | □ Eye drops | | | |
|  | □ I have no idea | □ I have no idea | | | |
| D2 | Which of the following are the symptoms of cataract and glaucoma?  (Select what you think is right) | | | | |
|  | **Cataract** | **Glaucoma** | |  | |
|  | □ Blurred vision | □ Blurred vision | | | |
|  | □ Eye pain | □ Eye pain | | | |
|  | □ Halo around the light | □ Halo around the light | | | |
|  | □ Nnausea | □ Nnausea | | | |
|  | □ Headache | □ Headache | | | |
|  | □ See things change in color | □ See things change in color | | | |
|  | □ Photophobia | □ Photophobia | | | |
|  | □ See black spots | □ See black spots | | | |
|  | □ Diplopia | □ Diplopia | | | |
|  | □ I have no idea | □ I have no idea | | | |
| D3 | Glaucoma can be cured. | | □True | □False | □Don’t know |
| D4 | Cataracts can cause glaucoma. | | □True | □False | □Don’t know |
| D5 | Excessive use of eyes can cause glaucoma. | | □True | □False | □Don’t know |
| D6 | The risk of glaucoma is lower without a family history. | | □True | □False | □Don’t know |
| D7 | There are no symptoms in the early stages of glaucoma. | | □True | □False | □Don’t know |
| D8 | In general, intraocular pressure below 30mmHg is in the normal range. | | □True | □False | □Don’t know |
|  |  | |  |  |  |
|  |  | |  |  |  |
| D9 | Which of the following is a risk factor for glaucoma? | |  |  |  |
|  | Age/aging | | □True | □False | □Don’t know |
|  | Genetic factors | | □True | □False | □Don’t know |
|  | High intraocular pressure | | □True | □False | □Don’t know |
|  | High myopia or farsightedness | | □True | □False | □Don’t know |
|  | Eye trauma | | □True | □False | □Don’t know |
|  | Long-term use of steroids | | □True | □False | □Don’t know |
|  | Diabetes | | □True | □False | □Don’t know |
|  | Migraine | | □True | □False | □Don’t know |
|  | Hypertension | | □True | □False | □Don’t know |
|  | Obesity | | □True | □False | □Don’t know |
|  | Lack of exercise | | □True | □False | □Don’t know |
|  | Nutritional imbalance | | □True | □False | □Don’t know |
|  | Too much exposure to digital devices such as cell phone, computer and TV | | □True | □False | □Don’t know |
|  | Lack of sleeping | | □True | □False | □Don’t know |

*～ PLEASE TURN THE PAGE AND CONTINUE～*

**Part 4: The status of glasses or contact lenses wearing**

E1 Do you **own** corrective glasses or contact lenses?

□ Yes (Skip To E2)

| E2 | □Have glasses  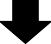 (Skip To E3~E5) |  | □Have contact lenses  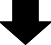 (Skip To E6~E8) |
| --- | --- | --- | --- |
| E3 | Reasons for wearing glasses (Choose one or more)  □Nearsightedness □Farsightedness □Presbyopia □Astigmatism  □Other____________________  How long have you had the glasses? ______ years | E6 | Reasons for wearing contact lenses (Choose one or more)  □Nearsightedness □Farsightedness □Presbyopia □Astigmatism  □Other____________________  How long have you had the contact lenses? ______ years |
| E4 | How often do you wear **glasses** on a daily basis? (When you are awake)  □Most of the time □Sometimes □Occasionally □Rarely | E7 | How often do you wear **contact lenses** on a daily basis? (When you are awake)  □Most of the time □Sometimes  □Occasionally □Rarely |
| E5 | What are the reasons that affect your frequency of wearing glasses? (Choose one or more) | E8 | What are the reasons that affect your frequency of wearing contact lenses? (Choose one or more) |
|  | □I feel dizzy or uncomfortable when wearing glasses. |  | □I feel dizzy or uncomfortable when wearing contact lenses. |
|  | □My vision is not improved much when wearing glasses. |  | □My vision is not improved much when wearing contact lenses. |
|  | □Wearing glasses is troublesome. |  | □Wearing contact lenses is troublesome. |
|  | □My vision has no problem in daily life. |  | □My vision has no problem in daily life. |
|  | □My glasses are broken or lost. |  | □My contact lenses are broken or lost. |

□No (Skip To E9)

| E9 | Has any ophthalmologist or optometrist advised you to get corrective glasses or contact lenses before?  □Yes (How long ago for the experience? ________ months ago) (Skip To E10)  □No (Skip To F1 in the next part) |
| --- | --- |
| E10 | What are the reasons that you do not get corrective glasses or contact lenses? (Choose one or more)   - I don’t have vision problems in daily life, there is no need to get a pair of glasses for now. - I don’t have time to get one / It is troublesome. - I can wear my family/friends’ glasses. - Glasses are too expensive. - I think my vision won’t be improved much after wearing glasses. - I may feel uncomfortable when wearing glasses. - Wearing glasses affects my outlook. - There are too many options of glasses that I am unable to understand. - I used to get a pair of glasses, but they were broken/lost. |

F1 Over the past a half year or longer, has any ophthalmologist (from hospital or clinic) advised you to have cataract surgery as soon as possible?

□Yes, I have undergone the surgery. (That’s the end of the interview)

□Yes, but I didn’t have the surgery. (Please continue to answer F2~F13)

□No, I haven’t visited an ophthalmologist/I didn’t have cataract at that time. (That’s the end of the interview)

□No, because the doctor believed that my cataract was not serious enough to require a surgery. (That’s the end of the interview)

| **We would like to know the reason you didn’t undergo cataract surgery.**  (Tick One On Each Line) | | | | |
| --- | --- | --- | --- | --- |
|  |  | 1.  It is an important factor. | 2.  It may be an important factor. | 3.  It is not an important factor. |
| F2 | Fear of blindness caused by failure of surgery. | □ | □ | □ |
| F3 | Even if the surgery would not cause blindness, I am worried that the surgery will cause sequelae or cataract recurrence. | □ | □ | □ |
| F4 | No time for surgery/My family has no time to accompany me for the surgery. | □ | □ | □ |
| F5 | I am worried that the recovery time would affect my living/work. | □ | □ | □ |
| F6 | I didn't know I had cataracts. | □ | □ | □ |
| F7 | I believe that my cataract is not serious enough to require surgery. | □ | □ | □ |
| F8 | There are too many kinds of cataract surgery and I am unable to understand, and/or I don’t have time to study on all options. | □ | □ | □ |
| F9 | I want to treat cataracts through other non-surgical methods such as applying eye drops, taking herbs, reducing sun exposure and so forth. | □ | □ | □ |
| F10 | Fear of pain | □ | □ | □ |
| F11 | I feel that my vision will not improve or the improvement is small after the operation. | □ | □ | □ |
| F12 | I am worried about the out-of-pocket expenses associated with the surgery | □ | □ | □ |
| F13 | Other resons _________________________________ | □ | □ | □ |

# To be filled by physician or medical staff

**Medical record number:** ___________ **Date of filling (yyyy/mm/dd):** _____ / ___ / ___

1. The patient has the following eye diseases:

□High myopia (above 500 degrees)　□High hyperopia (above 300)　□High astigmatism (above 250)

□Amblyopia　□Strabismus　□Keratoconus　□Corneal scars or degeneration

□Cataract without surgery (one eye)　□Cataract without surgery (both eyes)

□Glaucoma (one eye)　□Glaucoma (both eyes)　□Macular disease　□Retinal detachment

□Diabetic retinopathy　□Retinitis pigmentosa (night blindness)　□Optic nerve atrophy

2. Has this patient received the following eye surgery?

□Cataract surgery　□Retina surgery　□Cornea surgery　□Glaucoma surgery

□Eyeball trauma surgery　□Others (please specify) ________________

3. Does this patient have intraocular pressure measurement at this visit? □Yes □No

4. What is the intraocular pressure of the patient in most recent (or this) visit? ________mmHg □Don’t know

1. Does this patient need cataract surgery right now?

□Yes, and already delayed

□Yes, not delayed

□No

1. Physician-rated risk of glaucoma:

- High
- Medium
- Low
- Don’t know

□The patient already has glaucoma.

□Left eye □Right eye □Both eyes

1. Please given an overall assessment of the risk based on the following risk factors, please check relevant boxes:

□The patient is over age 40

□The patient has family history of glaucoma

□The patient has high [eye pressure](https://www.aao.org/eye-health/anatomy/eye-pressure)

□The patient is [farsighted](https://www.aao.org/eye-health/diseases/hyperopia-farsightedness) or [nearsighted](https://www.aao.org/eye-health/diseases/myopia-nearsightedness)

□The patient had an [eye injury](https://www.aao.org/eye-health/tips-prevention/injuries)

□The patient is using long-term steroid medications

□The patient has [corneas](https://www.aao.org/eye-health/anatomy/cornea-103) that are thin in the center

□The patient has thinning of the optic nerve

□The patient has [diabetes,](https://www.aao.org/eye-health/tips-prevention/diabetes) and high [blood pressure](https://www.aao.org/eye-health/anatomy/blood-pressure)

1. How many times have you seen this patient?

□ First time □ 2-3

□ 4-5 □ >=5

　　　　　　　　　　　　Signature：_______________
