# Supplementary material for: Accuracy of perceived glaucoma risk by patients in a clinical setting
Source: PLoS One. 2021 Sep 16;16(9):e0257453. doi: 10.1371/journal.pone.0257453 (PMC8445404; doi:10.1371/journal.pone.0257453)
Supplement: Attachment — Submitted filename: R1-Response letter.docx [file pone.0257453.s003.docx]

Dear Editor and Reviewer,

Thank you for the valuable comments on our manuscript. They have indeed helped us enhance our manuscript. We have revised the manuscript based on these comments. All changes are tracked in a different color.

Reviewer #1: Dear authors,

congratulations to your great work! The data emphazise the impact of education about glaucoma disease in the population.
Only some minor points have to be revised:

table 2: "knowledge"

**Response:** This has been corrected in the revised version

table 3: "Glaucoma" instead of "galaucoma"

**Response:** Thank you, this has been corrected in the revised version

The Figures Legends:
Please describe the figures and their results in more detail in order that the readers will understand the main findings.

**Response:** More detailed figure legends have been provided in the revised version.

Journal comments:

1. Please ensure that your manuscript meets PLOS ONE's style requirements, including those for file naming. The PLOS ONE style templates can be found at 
https://journals.plos.org/plosone/s/file?id=wjVg/PLOSOne_formatting_sample_main_body.pdf and 
https://journals.plos.org/plosone/s/file?id=ba62/PLOSOne_formatting_sample_title_authors_affiliations.pdf

**Response:** Formatting has been checked.

2. Please review your reference list to ensure that it is complete and correct. If you have cited papers that have been retracted, please include the rationale for doing so in the manuscript text, or remove these references and replace them with relevant current references. Any changes to the reference list should be mentioned in the rebuttal letter that accompanies your revised manuscript. If you need to cite a retracted article, indicate the article’s retracted status in the References list and also include a citation and full reference for the retraction notice.

**Response:** The reference list has been checked for accuracy.

3. Please include in your Methods section (or in Supplementary Information files) the participating hospitals/institutions.

**Response:** The hospital name has been added in the revised version.

Please include additional information regarding the survey or questionnaire used in the study and ensure that you have provided sufficient details that others could replicate the analyses. For instance, if you developed a questionnaire as part of this study and it is not under a copyright more restrictive than CC-BY, please include a copy, in both the original language and English, as Supporting Information.

**Response:** The questionnaire has been included in this submission.

Please provide additional information regarding the participant eligibility criteria used and how participants were recruited for the study.

**Response:** Details on participant eligibility criteria have been provided in the revised version.

4. Please include your full ethics statement in the ‘Methods’ section of your manuscript file. In your statement, please include the full name of the IRB or ethics committee who approved or waived your study, as well as whether or not you obtained informed written or verbal consent. If consent was waived for your study, please include this information in your statement as well.

**Response:** Detailed IRB information has been provided in the revised version.

5. Please include a copy of Table 1b which you refer to in your text on page 10.

**Response:** Sorry about the typo. It should have been “Figure 1b”.
